# Anti-immigrant Rhetoric and the Experiences of Latino Immigrants in the Emergency Department

**DOI:** 10.5811/westjem.2021.2.50189

**Published:** 2021-05-07

**Authors:** Carolina Ornelas, Jacqueline M. Torres, Jesus R. Torres, Harrison Alter, Breena R. Taira, Robert M. Rodriguez

**Affiliations:** * University of California, San Francisco, Department of Emergency Medicine, San Francisco, California; † University of California, San Francisco, Department of Epidemiology and Biostatistics, San Francisco, California; ‡ Highland Hospital - Alameda Health System, Department of Emergency Medicine, Oakland, California; § Olive View - UCLA Medical Center, Department of Emergency Medicine, Sylmar, California

## Abstract

**Introduction:**

Anti-immigrant rhetoric and increased enforcement of immigration laws have induced worry and safety concerns among undocumented Latino immigrants (UDLI) and legal Latino residents/citizens (LLRC), with some delaying the time to care.^1^ In this study, we conducted a qualitative analysis of statements made by emergency department (ED) patients – a majority of whom were UDLI and LLRC – participating in a study to better understand their experiences and fears with regard to anti-immigrant rhetoric, immigration enforcement, and ED utilization.

**Methods:**

We conducted a multi-site study, surveying patients in three California safety-net EDs serving large immigrant populations from June 2017–December 2018. Of 1684 patients approached, 1337 (79.4%) agreed to participate; when given the option to provide open-ended comments, 260 participants provided perspectives about their experiences during the years immediately following the 2016 United States presidential election. We analyzed these qualitative data using constructivist grounded theory.

**Results:**

We analyzed comments from 260 individuals. Among ED patients who provided qualitative data, 59% were women and their median age was 45 years (Interquartile range 33–57 years). Undocumented Latino immigrants comprised 49%, 31% were LLRC, and 20% were non-Latino legal residents. As their primary language, 68% spoke Spanish. We identified six themes: fear as a barrier to care (especially for UDLI); the negative impact of fear on health and wellness (physical and mental health, delays in care); factors influencing fear (eg, media coverage); and future solutions, including the need for increased communication about rights.

**Conclusion:**

Anti-immigrant rhetoric during the 2016 US presidential campaign contributed to fear and safety concerns among UDLI and LLRC accessing healthcare. This is one of the few studies that captured firsthand experiences of UDLI in the ED. Our findings revealed fear-based barriers to accessing emergency care, protective and contributing factors to fear, and the negative impact of fear. There is a need for increased culturally informed patient communication about rights and resources, strategic media campaigns, and improved access to healthcare for undocumented individuals.

## INTRODUCTION

Anti-immigrant rhetoric in the 2016 United States presidential campaign and subsequent statements made and enforcement actions taken by the 45th US president have had a substantial impact on undocumented Latino immigrants’ (UDLI) feelings of safety and healthcare access.^1^ Healthcare staff in clinics noted increased fear of deportation and family separation among their UDLI patient populations (especially among mixed status families) and reduced utilization of healthcare and social services.^2^ Providers also noted a “chilling effect,” where individuals were not exercising legitimate rights, such as reporting crimes and accessing reproductive healthcare, due to fear of identification as a public charge and negative repercussions on immigration applications.^3^,^4^,^5^

Healthcare setting and place act upon the political and policy landscape, impacting the health and healthcare of immigrants and their family members. Some states have increased protections and access for immigrants, while others have introduced barriers. For example, California, New York, and Washington allow legal permanent residents access to Medicaid immediately, instead of the standard five years after legalization. Other states, such as Alabama and Arizona, restrict access to social and medical services for non-legalized immigrants.^6^ In addition to the state-by-state landscape, there are federal policies that influence the lives and healthcare of immigrants in the US. For example, the Emergency Medical Treatment and Labor Act (EMTALA) mandates that anyone, regardless of insurance or legal status, can access care in the emergency department (ED).^1^

While other qualitative studies have documented the challenges of living as an undocumented person,^7^ and general barriers to healthcare,^2^,^4^,^8^ there is limited literature examining the firsthand experience of undocumented immigrants in the emergency care system (an important safety net and primary health access point), especially in a time of recent heightened immigration enforcement and anti-immigrant rhetoric. In our prior quantitative research, we found that undocumented ED patients linked the anti-immigrant rhetoric surrounding the 2016 US presidential election to increased fear of accessing healthcare services, with some undocumented patients describing increased delays in accessing emergency care as a result.^1^ In the present qualitative analysis of these patients’ perspectives we aimed to provide further nuance and details regarding the experience of undocumented patients in the ED, including the fear of accessing emergency care, by surveying patients during real-time ED care.

## METHODS

### Ethics Statement

We obtained institutional review board (IRB) approval from the University of California of San Francisco Committee on Human Research, the Olive-View UCLA Medical Center Education and Research Institute, and the Highland Hospital—Alameda Health System IRB to conduct this survey study. We obtained scripted, verbal consent from participants and collected qualitative data on a survey form with no identifying information.

**Population Health Research Capsule**

What do we already know about this issue?

*Anti-immigrant rhetoric and increased immigration enforcement have induced fear and delays in emergency care among undocumented Latino immigrants (UDLI).*

What was the research question?

*How do UDLI experience the fear surrounding anti-immigrant rhetoric and emergency department utilization?*

What was the major finding of the study?

*There was a wide range of fears and modifying factors, which drove down access to care and perceived health.*

How does this improve population health?

*Culturally informed communication about rights and resources, and addressing structural barriers, can reduce fear and facilitate access to emergency care for UDLI.*

### Study Design and Setting

From mid-June 2017 to mid-December 2018, we conducted a survey study at three urban county hospitals in California. At these EDs, 45.3% of visits were by patients of self-declared Latino ethnicity in 2017. Methodological details and quantitative results have previously been reported.^1^ Briefly, patients were recruited upon presentation to the ED using a convenience sampling method. Patients were excluded if they met any of the following characteristics: 1) trauma; 2) transfer from another facility; 3) inability to participate in an interview because of intoxication, altered mental status, or critical illness; 4) incarceration; and 5) on psychiatric hold. All patients who met inclusion criteria were approached by trained bilingual research personnel.

The quantitative survey questions inquired about anti-immigrant rhetoric and fear and safety concerns. Questions included the following: “Did any of these statements [eg, the president wants to build a wall, the president wants to deport immigrants, or the president wants to prevent immigrants from getting healthcare] make you afraid to come to the emergency department?”; “When thinking about going to the doctor or ER for a health problem, do you feel more worried or scared about getting identified as an undocumented immigrant NOW compared to how you felt ONE YEAR AGO?”; and “Have these statements made you feel worried or unsafe living in the US?”. To more deeply understand patient experiences and perspectives, we provided participants with the option to provide open-ended comments after completing the survey, asking, “Do you have additional comments, including about the study or survey questions?”. The present study is based on responses to this final open-ended question. Study personnel documented patient comments through a combination of direct quotes and their own summarization. We did not collect audio recordings to protect patient privacy and confidentiality.

### Data Management

Across the three sites, researchers documented a total of 574 open-ended patient commentary entries among the 1318 total surveys collected in the study. We excluded a total of 314 comments because the text consisted of researcher notes about the interview itself, clarifications about the quantitative survey responses, and patient stories irrelevant to immigrant health or the ED experience. We included a total of 281 open-ended responses in our analysis. We consolidated 21 responses that had overlapping participant identifications (i.e., were for the same person surveyed more than once in the ED). These data were consolidated into a total of 260 entries from 260 individual survey respondents (Figure).

### Data Analysis

We analyzed the qualitative data using constructivist grounded theory, which combines deductive and inductive thematic analysis.^9^ The analysis was driven by the research question: What are patients’ experiences and fears with regard to anti-immigrant rhetoric, immigration enforcement, and ED utilization? We used predetermined survey domains, such as fear of accessing care, and added thematic categories to capture the dimensions of fear, including protective and contributive factors. One investigator (CO) coded all comments. A second investigator (JMT) coded a random subsample of 87 (one third) comments to ensure consistency and replicability of coding. Among the 260 comments, there were 213 distinct codes, grouped into six broad themes.

## RESULTS

### Demographics

Of the 260 individuals included in the qualitative study, 41% were men and 59% were women. Their median age was 45 years (interquartile range, 33–57). Undocumented Latino immigrants (UDLI) comprised 49%, 31% were legal Latino residents (LLRC), and 20% were non-Latino legal residents (NLRC). Spanish language was the primary language for 68%, and 32% spoke English as their primary language (Table 1). Compared to our quantitative results, which were previously reported,^1^ respondents to our qualitative study had a similar median age and primary language distribution, but greater proportions of UDLI and women.

### Grounded Theory Analysis

Of the following themes, factors modifying fear, fear as a barrier to care, and impact of fear on health and healthcare were most frequently mentioned.

#### Theme 1: Fear as a barrier to care (frequency: 32%)

Although all ED patients in the study eventually sought care, some UDLI and a few LLRC noted feeling afraid before visiting the hospital. Others, especially LLRC, shared that undocumented family and friends were afraid to visit the ED. Fears ranged from family separation, negative consequences on future legalization (ie, public charge^5^), the potential for discrimination and denial of services, and encountering law enforcement in the hospital. Other barriers included healthcare expenses, long wait times, and language barriers. Nevertheless, not all expressed fear of visiting the hospital.

#### Theme 2: Factors modifying fear (frequency: 38%)

Factors that increased fear, especially among UDLI, included exposure to media coverage of immigration enforcement, seeing deportations within the community, and the political climate. Factors that decreased fear included knowledge of one’s rights, less media coverage of immigration issues, positive healthcare experiences, and having health insurance. Fear also varied by place and time. Many mentioned feeling safe in a sanctuary city, in California, and in their respective hospitals, while highlighting negative experiences for loved ones in states outside of California.

The presence and perception of law enforcement was a commonly cited reason that influenced fear. A couple of individuals believed that hospitals collaborate with immigration enforcement and that providers report patients. This suspicion was confirmed by seeing law enforcement outside or in the hospital. For some, fear of interacting with law enforcement was a barrier to reporting incidences of domestic violence in the context of ED care. Individuals who did not believe hospitals comply with immigration enforcement cited this as a reason to not have fear.

#### Theme 3: Impact of fear on health and healthcare (frequency: 16%)

Fear had a negative impact on some participants’ health. The stress and worry of immigration enforcement were all consuming, and they felt worried or “on edge” all the time. Some endorsed worsening headaches, increased feelings of anxiety, and elevated/uncontrolled blood pressure. The majority of respondents, however, were not directly impacted and instead recounted how the fear impacted their friends, family, and neighbors.

Sometimes this fear led to a delay in seeking care. Reasons for delaying care were mistrust and misinformation around reporting, deportations, and discrimination within hospitals. For individuals who did not delay, they cited reasons including medical necessity (pain, “felt like I was going to die”). A group of patients shared stories of themselves or individuals they knew who experienced morbidity and mortality from delaying care for emergent and serious conditions including appendicitis, end stage renal disease requiring dialysis, infections, and a retinal detachment.

#### Theme 4: Effect on the broader community (frequency: 4%)

Although the majority of comments were from UDLI, LLRC and NLRC commented on how they were also impacted by anti-immigrant rhetoric. Legal Latino residents/citizens expressed fear of losing their rights and being persecuted for their race/ethnicity. One mother noted her child crying over having to move back to Mexico after the election, despite being legal residents. On three separate occasions, participants highlighted that “anything can happen.” Others noted an increase in explicit racism during the time period following the 2016 presidential election targeting people of color who were perceived as foreign.

#### Theme 5: Coping strategies and protective factors (frequency: 7%)

Although fearful, worried, and anxious, individuals developed coping strategies and found sources of strength and resilience within themselves and their communities. Coping strategies included avoidance (eg, not looking at the news), acceptance (eg, the possibility of deportation), and problem solving (eg, taking legal rights courses, leaving the US). The main protective factor was having knowledge and information about one’s rights. Individuals learned about their rights through local churches, community clinics, hospital staff, and media sources.

#### Theme 6: Potential future interventions in the ED (frequency: 3%)

Several individuals shared suggestions for future ED changes and interventions that could help mitigate fear, including having more staff who spoke Spanish and identified as part of the Latino community, increasing communication about one’s rights, and clarifying the role of law enforcement in the hospital through television, advertisement, and billboard messages. Exemplar quotes for the six themes are presented in Table 2.

## DISCUSSION

Anti-immigrant rhetoric and heightened immigration enforcement surrounding the campaign and results of the 2016 US presidential election has been linked to increased worry and safety concerns among undocumented individuals. Providers have noted delays in care, reduced utilization of healthcare and social services, and fewer individuals accessing legal rights and resources; these impacts have also been documented in a growing number of studies.^1^–^4^ For example, there is an expanded definition of a “public charge,” wherein certain individuals applying for a green card (permanent resident card) or visa could be denied for using government resources such as Medicaid and housing assistance; although it does not apply to all immigrants, this has instilled trepidation about accessing resources even among immigrants who are not affected by public charge.^5^ Given the ED’s role as a primary source of care for many patients and that delays in care for conditions requiring the ED can be life-threatening, we further examined the perspectives of UDLI in a multi-site study at three safety-net EDs with large immigrant populations, collected with respect to the 2016 US presidential nomination.

We found that fear related to immigration status can serve as a barrier to ED care for patients, especially for undocumented immigrants. The fear impacted a variety of individuals, led to delays in care, negatively contributed to perceptions of physical and mental health, and was influenced by factors such as knowledge of one’s rights and media coverage. The fear among UDLI respondents is consistent with other studies,^2^,^4^,^7^,^8^ although none have been specific to the ED. Our study also discovered fear among legal Latino residents, supporting a recent study showing growing deportation fear among Latino US citizens.^10^ A growing body of research suggests that living in fear contributes to chronic stress, which is associated with increased risk for mental health conditions (eg, depression), and chronic diseases (eg, heart disease and diabetes).^11^,^12^ Addressing fear, especially within important sources of healthcare such as the ED, is critical for the health and wellness of our immigrant communities, and is essential in the context of heightened immigration enforcement and anti-immigrant rhetoric.

Patients suggested that one approach to addressing fear is educating patients about their rights, inside and outside of the hospital, and even in sanctuary settings. The single most cited factor that mitigated fear was knowing one’s rights. While individuals learned about their rights through local churches, community clinics, hospital staff, and media sources, they expressed a need for further education. Emergency departments and hospitals could collaborate with and build on existing, trusted community efforts. Especially in times of the COVID-19 pandemic where in-person outreach is limited, media and virtual efforts may play a crucial role in healthcare systems’ communication about rights for immigrant patients. However, it is important that these efforts are strategic, and mindful of UDLI experiences and concerns. For our participants, the media played a dual role of contributing to fear through coverage of immigration enforcement and alleviating fear through education and empowerment. Given the misconceptions about the role of law enforcement and providers in the hospital, communication efforts aimed at reducing fear among immigrant patients should address these roles, as well as clarify existing protections against discrimination and denial of services (eg, EMTALA).

Finally, there is a need to confront structural barriers in our healthcare system for UDLI. Beyond fear of discovery, UDLI mentioned other barriers to care, mainly healthcare expenses. There are large gaps in health insurance eligibility and enrollment for UDLI in the US, with eligibility largely limited to select private, state, and county-specific options^13^; lack of insurance is a substantial barrier to care. Finally, some individuals only felt safe at specific hospital sites or clinics. It is important to understand and build structural interventions and formulate policies that contribute to these feelings of safety and trust more broadly.

## LIMITATIONS

We note several limitations, including the convenience sample of the overall study and the fact that a relatively small percentage of participants provided qualitative data. As compared to the larger quantitative study, the demographic characteristics of qualitative study participants were substantially different. Comments represented largely middle-aged, Spanish-speaking individuals, of whom half were ULDI and a third were LLRC. Although skewed with respect to the larger quantitative study, this analysis highlights the experiences of the two communities impacted the most by anti-immigrant rhetoric.^1^

In addition, our study captured the experiences of individuals who ultimately sought emergency care. To directly represent the experiences of UDLI who completely avoid the ED due to fear and other barriers, future study sites may include other locations such as community-based organizations and clinics. Also, our study occurred in sanctuary cities within a sanctuary state and does not reflect the experiences of all patients in different settings but may reflect perspectives of hospitals with large immigrant populations.

Other limitations included the lack of recordings or follow-up to elicit further information, a decision we made to maintain confidentiality and security. However, given the opening for unstructured commentary, participants shared a wide range of experiences and perspectives that were not captured in our quantitative data, and to mediate recording error, research assistants documented notes right after the interview.

## CONCLUSION

In a qualitative analysis of ED patients’ perceptions of safety and emergency care in the years following the 2016 US presidential election, we found that fear played a substantial role in experiences with accessing emergency care.^1^ Some patients described decremental impacts on their mental and physical health and delays in care due to fear of discovery. This fear was not limited to undocumented Latino immigrants, affecting also legal Latino residents. Patients coped through avoidance, acceptance, and problem solving, including learning about their rights, and identified communication about rights as a key future intervention. Our study supports the need for the following: 1) increased culturally and linguistically appropriate patient communication, including media campaigns, about rights/resources and the role of law enforcement and healthcare providers; and 2) efforts within and outside the ED to address structural barriers to emergency care for UDLI.

## Figures and Tables

**Figure:**
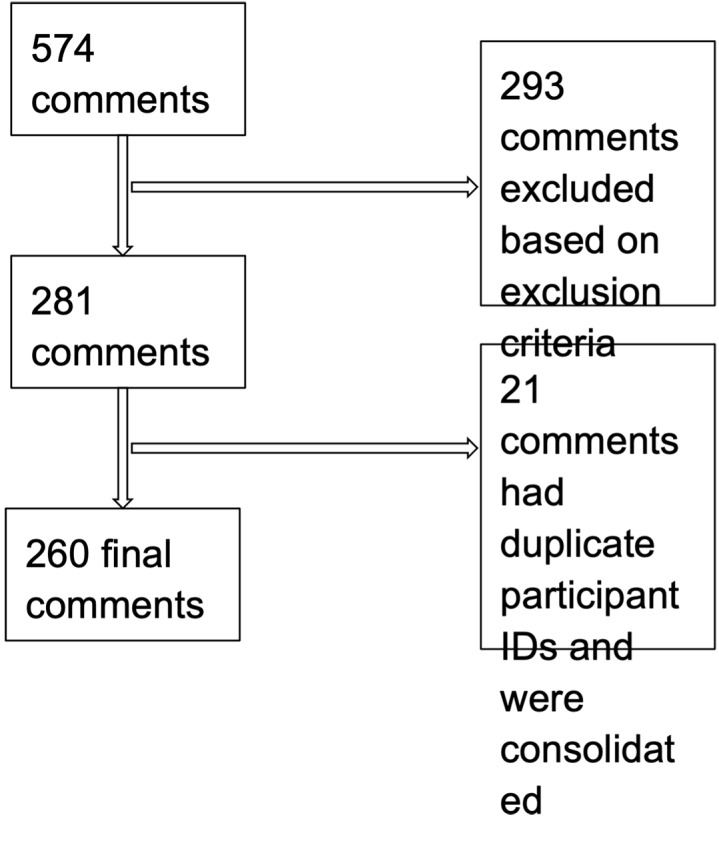
Process of excluding and consolidating open-ended patient commentary for multi-site emergency department study.

**Table 1:** Demographic information of multi-site emergency department survey participants with open-ended commentary.

	UDLI n (%)	LLRC n (%)	NLRC n (%)
Total number	129 (49%)	80 (31%)	51 (20%)
Gender			
Men	47 (36%)	41 (51%)	20 (39%)
Women	82 (64%)	39 (49%)	31 (61%)
Median age in years (IQR)	42 (32 to 54)	48 (38 to 59)	45 (30 to 57)
Primary language			
English	7 (5%)	27 (34%)	45 (88%)
Spanish	122 (95%)	53 (66%)	0 (0%)
Other	0 (0%)	0 (0%)	6 (12%)

*UDLI*, undocumented Latino immigrants; *LLRC*, legal Latino residents/citizens; *NLRC*, non-Latino legal residents; *IQR*, interquartile range.

**Table 2:** Six themes with exemplar quotes from analysis of emergency department patient perspectives.

Theme	Exemplar quote
Experiencing fear	“I’ve been worried now with everything going on with public charge, it puts me in a hard spot because I am very sick but now I hear I might not be able to get papers...I don’t know whether seeking medical care is going to prevent me from renewing.” “My mom and dad (who are undocumented) are scared to come to the ER because of getting a bill...I really feel like this is the main reason a lot of undocumented people don’t come in.”
Factors modifying fear	“I’ve seen my regular doctor (this past year) but not come here because it’s different here, having to pass through security. I’ve been afraid that I might get reported.” “Was not afraid to come to the ER because [I] had gotten “know your rights training” at a primary care clinic.”
Impact of fear	“I get sick, I feel so sick from the worrying and the stress, worrying about my family. I get headaches all the time now, and nerves all the time.” “My nephew who needs dialysis but he didn’t have coverage [due to documentation status], and so he ended up leaving to Tijuana 3 weeks ago because he couldn’t get it here. I told him to come here, but he said no, better go to Mexico. When he got there he ate some tacos and started vomiting blood and so they took him to the hospital and they told him one kidney was completely dead, and the other had 18% function. He’s only 24.”
Effect on the broader community	“Even though [I’m a Latino] resident, this current administration makes [me] scared to seek care because anything could happen.” “Though lots of the negativity of immigration is directed towards Mexicans, people of other backgrounds are also treated so poorly... a white woman [was] cursing out a Thai woman and telling the Thai woman to go back to Thailand.
Coping strategies and protective factors	“On the news there are announcements on how people should not be afraid to go get services, including going to the doctor.” “In (her primary care clinic) they gave me the red card and oriented me to my rights, that it’s ok to come here, what to do if immigration comes to my door.”
Potential future interventions	“It should be announced to everyone that the police department is here only to keep peace. I sent my partner home because I was scared that they were going to arrest us.” “On TV there are so many bad news stories, it would be helpful to have announcements or ads that the public hospitals are not affected (by Trump), that people can keep using them and feel safe.”

*ER*, Emergency Room
